# Modulatory Effects of Heat-Inactivated Streptococcus Thermophilus Strain 7 on the Inflammatory Response: A Study on an Animal Model with TLR3-Induced Intestinal Injury

**DOI:** 10.3390/microorganisms11020278

**Published:** 2023-01-20

**Authors:** Gilbert Aaron Lee, Yu-Wei Chang, Wan-Li Lin, Yu-Chen S. H. Yang, Wei-Jen Chen, Fu-Huan Huang, Yun-Ru Liu

**Affiliations:** 1 Department of Medical Research, Taipei Medical University Hospital, Taipei City 110, Taiwan; 2 Department of Microbiology and Immunology, School of Medicine, College of Medicine, Taipei Medical University, Taipei City 110, Taiwan; 3 Child Development Research Center, Taipei Medical University Hospital, Taipei City 110, Taiwan; 4 TMU Research Center for Digestive Medicine, Taipei Medical University, Taipei City 110, Taiwan; 5 Joint Biobank, Office of Human Research, Taipei Medical University, Taipei City 110, Taiwan; 6 Syngen Biotech International, Shah Alam 40460, Malaysia; 7 Division of Pediatric Surgery, Department of Surgery, Taipei Medical University Hospital, Taipei City 110, Taiwan

**Keywords:** *Streptococcus thermophilus*, microbiota, poly I:C, inflammation, IL-15

## Abstract

Rotavirus infections result in severe gastroenteritis with a detrimental inflammatory response in the intestine. Because probiotics have an anti-inflammatory effect and can modulate the gut microbiota profile, they can be used as a biotherapy for inflammatory intestinal diseases. In this study, we isolated *Streptococcus thermophilus* strain 7 (ST7) from cow milk and examined the effect of heat-inactivated ST7 on the intestinal histopathological score, inflammatory cytokine levels, T-cell activation and effector function, and microbiome profile in a mouse model with intestinal injury induced by polyinosinic-polycytidylic acid (poly I:C), a Toll-like receptor 3 agonist. The results indicated that ST7 treatment prevented weight loss and intestinal injury and prevented the upregulation of serum interleukin-6 (IL-6), tumor necrosis factor-α, and IL-15 levels in intestinal epithelial cells; prevented the upregulation of inflammation-associated Gammaproteobacteria and *Alistipes*; and increased the levels of Firmicutes in fecal microbiota after poly I:C stimulation. ST7 treatment also increased the serum interferon-γ (IFN-γ) level and promoted the expression of IFN-γ in both CD8 and CD4 T cells. In summary, ST7 prevented the inflammatory response, promoted the T-cell effector function, and modulated the microbiota profile of mice with poly I:C-induced small intestine injury.

## 1. Introduction

Rotavirus infections are a leading cause of severe gastroenteritis in children [1]. Rotaviruses are double-stranded RNA (dsRNA) viruses that stimulate inflammatory responses in the intestine [2,3] and alter the function of the small intestine epithelium, resulting in diarrhea. The destruction of enterocytes results in primary malabsorption, decreased digestion, and acute diarrhea [3]. Enterocytes distinguish microbial antigens through pattern recognition receptors, such as Toll-like receptors (TLRs), which play a critical role in innate immunity by facilitating the identification of the genomic dsRNA of several viruses or dsRNA synthesized during viral replication. Polyinosinic-polycytidylic acid (poly I:C) is a synthetic analog of retroviral genomic dsRNA. It stimulates TLR3, which in turn activates nuclear factor-κB and induces the production of type I interferon (IFN) [4]. In a poly I:C-induced intestinal injury animal model, the intraperitoneal administration of poly I:C triggered a local intestinal immune response and caused severe mucosal damage in the gut in a TLR3-dependent manner, and, neutralizing the effects of interleukin-15 (IL-15), protected against villous damage and prevented weight loss [5]. Taken together, these findings indicate that TLR3 and IL-15 are critical factors that result in intestinal inflammation and injury.

TLR3 mediates leukocyte recruitment and activation through inflammatory cytokines and chemokines [6]. After poly I:C stimulation, the duodenum is usually the organ most affected by intestinal mucosal injury. Poly I:C stimulation also induces the oversecretion of the inflammatory factors tumor necrosis factor-α (TNF-α) and IL-6 by intestinal tissue, mesenteric lymph nodes, and spleen immune cells [4,6,7,8]. Providing an effective immune response against viral infections depends on the activation of cytotoxic T cells, which can kill virus-infected cells. Activated CD4^+^ T cells can activate dendritic cells to properly activate naïve CD8^+^ T cells, which then differentiate into cytotoxic T cells that release cytotoxic granules and IFN-γ. IFN-γ is essential in the immune response to various viral infections because it can induce an antiviral state in uninfected cells and can enhance the cytotoxic function of CD8^+^ T cells [9]

The intestinal mucosa comprises intestinal epithelial cells (IECs), intestinal immune cells, and the intestinal microbiota, all of which play vital roles in gut immunity and in the detection of and response to gut stimuli. Under normal conditions, IECs and immune cells interact, respond to external stimuli, and maintain intestinal stability. The gut microbiota is a complex ecosystem consisting of trillions of bacteria that live in the digestive tract of humans and other animals [10]. A healthy gut microbiota plays a key role in promoting and maintaining a balanced immune response [11]. During pathogenic infections, the gut microbiota, immune cells, and IECs are activated to combat invading bacteria and viruses [12].

Oral probiotics have anti-inflammatory effects and can modulate the gut microbiota profile [13,14,15]. In lipopolysaccharide (LPS, TLR4 agonist)-induced septic mouse models, oral live *Streptococcus thermophilus* treatment ameliorates inflammation and intestinal injury and modulates the microbiota profile [16]. Although probiotics have antipathogenic and immunomodulatory properties [17], the underlying mechanisms of TLR3-induced inflammation in the intestine remain unclear.

Generally, the intake of live intact microorganisms poses risks of mutations and intrinsic resistance, which in turn results in the development of antibiotic-resistant phenotypes [18]. Advancements in the field of probiotics have facilitated the replacement of live intact microorganisms with heat-inactivated structural components [18]. According to a number of studies, heat-inactivated bacteria play a role in the modulation of immune responses and in the maintenance of intestinal barrier integrity [18,19]. However, the effect of heat-inactivated bacteria on the modulation of inflammatory responses and the microbiota profile in gastroenteritis remains unclear. In this study, we investigated whether heat-inactivated *S. thermophilus* strain 7 (ST7) can be used as a preventive treatment for modulating the intestinal inflammatory response, T-cell effector function, and microbiota profile of a TLR3-induced small intestine injury animal model. We also explored whether the functional effects of heat-inactivated ST7 on intestinal inflammatory diseases can aid in the preventive treatment of gastroenteritis.

## 2. Materials and Methods

### 2.1. Microorganisms

Strain ST7 (Syngen Biotech, Taipei City, Taiwan) was anaerobically isolated from a single colony cultured from cow milk. After ST7 enumeration, the ST7 bacteria in the culture suspension were then boiled at 70 °C for 30 min to obtain heat-killed ST7. The supernatant was then discarded, the heat-killed ST7 bacteria were resuspended in saline, and the concentration was adjusted to 10^9^ CFU/mL.

### 2.2. Enumeration of ST7

The number of ST7 bacteria in the culture suspension was determined using a previously reported method [20]. Briefly, 1 mL of the live ST7 culture suspension was serially diluted in a 0.1% peptone diluent. The diluted culture suspension was then grown on an M17 agar plate (Sigma, USA) for 48 h at 37 °C. Finally, the plates containing 25–250 colonies were enumerated, and the counts are presented as the CFU/mL of the culture suspension.

### 2.3. Oral Heat-Killed ST7 Experiment

In this study, a poly I:C-induced intestinal inflammatory mouse model was adapted from a previous study [5], and the functional effect of heat-killed ST7 was examined. Male 8-week-old C57BL/6JNarl mice were obtained from the National Laboratory Animal Center, Taiwan. All mice were bred in our facility under specific pathogen-free conditions and were maintained under a 12 h light/dark cycle. All mice were fed a conventional balanced diet ad libitum. ST7 cells (10^7^ cells/mouse/day) were orally administered to the mice for 7 days. On day 8, both ST7-treated mice and control mice (treated with drinking water) were weighed, and they then received an intraperitoneal dose (100 μg/g body weight in phosphate-buffered saline [PBS]) of poly I:C (Sigma). Their spleens, mesenteric lymph nodes, and blood were collected 2 h later for the cytokine and immune cell activation profiles analysis. In another set of experiments, the small intestine and feces were collected 24 h after stimulation with poly I:C. The feces were stored at −80 °C for gut microbiota analysis. The small intestines were examined for intestinal histology and IL-15 expression. All experimental procedures were performed in accordance with the LAC-2020-0091 protocol, which was approved by the Institutional Animal Care and Use Committee of Taipei Medical University, and in strict accordance with the ARRIVE guidelines.

### 2.4. Intestinal Histopathological Evaluation

Small intestine samples were excised, washed with PBS, and immersed in a formalin solution. Once fixed, the samples were dehydrated, embedded, cut into 4 μm thick serial sections, and stained with hematoxylin and eosin (H&E) for examination under a light microscope. All slides were coded and evaluated in a blinded manner in sections. Histopathologic scores and villus lengths were evaluated as described previously [21,22]. A summary of histopathologic scores was indicated by the following description. Score 0: normal ileum with intact epithelium; Score 1: mild mucosal inflammatory cell infiltrate; Score 2: mild diffuse inflammatory cell infiltrate in mucosa and submucosa; Score 3: moderate inflammatory cell infiltrates in mucosa and submucosa with villous blunting.

### 2.5. Immunohistochemistry

To perform an immunohistochemical analysis, intestine samples were fixed in formalin for 3 days, and 0.5 cm coronal slices were embedded in paraffin and cut into 4 µm thick sections [23,24]. The sections were then deparaffinized, rehydrated, and subjected to an antigen retrieval process and then stained with an anti-IL-15 antibody (R&D Systems, Minneapolis, MN, USA), followed by incubation with a peroxidase-conjugated AffiniPure Donkey Anti-Goat IgG antibody (Jackson ImmunoResearch Laboratories, West Grove, PA, USA). Subsequently, the sections were stained with 3,3′-diaminobenzidine and hematoxylin (BioLegend, San Diego, CA, USA) and were observed under a microscope (Olympus BX43; Olympus, Tokyo, Japan). IL-15-positive areas were quantified using HistoQuest tissue analysis software (TissueGnostics, Vienna, Austria).

### 2.6. Serum Cytokine Concentrations

Serum TNF-α, IL-6, and IFN-γ concentrations were measured using commercially available enzyme-linked immunosorbent assay (ELISA) kits in accordance with the manufacturer’s recommendations (BioLegend and R&D Systems). The range of the standard curves was between 7.8 and 500 pg/mL.

### 2.7. 16S Ribosomal Ribonucleic Acid Gene and Next-Generation Sequencing

The detailed procedures for the high-throughput sequencing of the 16S ribosomal ribonucleic acid (rRNA) gene and next-generation sequencing are described in our previous study [25]. The mice were allowed to defecate freely in clean cages, and DNA was extracted from fresh stool samples by using a QIAamp Fast DNA Stool Mini Kit (Qiagen, Hilden, Germany). Library preparation was conducted using an Illumina MiSeq system in accordance with the protocol for 16S rRNA gene amplicons. Universal primers (341F and 805R) were used to amplify the V3–V4 region of the bacterial 16S rRNA genes. Demultiplexed, paired reads were removed using Cutadapt (v1.12). Filtered reads were processed using the DADA2 package (v1.14.1) in R software (v3.6.1) [26,27]. However, the rarefying procedure was not performed. The V3–V4 sequence variants in the samples were inferred using the DADA2 package, and the frequency of each sequence variant in each sample was determined. The taxonomy assignment was conducted using the SILVA database (v138) [28], with a minimum bootstrap confidence of 80. The multiple sequence alignment of variants and phylogenetic tree preparation were performed using DECIPHER (v2.14.0) and the phangorn package (v2.5.5), respectively [29]. Taxonomy assignment, count tables, and phylogenetic trees were applied to a phyloseq object, and community analysis was performed using phyloseq software (v1.30.0) [30]. Alpha diversity indices were calculated to estimate the richness function of the phyloseq package. All statistical analyses were performed using the Wilcoxon–Mann–Whitney test (α = 0.05). To assess community dissimilarity between the groups, UniFrac distances were calculated using the GUniFrac package (v1.1) [31]. Principal coordinate analysis (PCoA) ordination was applied for UniFrac distances, and the adonis and betadisper functions from the vegan package (v2.4) of R were used to analyze the dissimilarity of compositions between the groups and the homogeneity of their dispersion, respectively.

### 2.8. Flow Cytometry

Spleens and mesenteric lymph nodes were collected from the control, poly I:C-treated, ST7-treated, and polyI:C+ST7-treated mice. All tissues were ground using a Wheaton tissue grinder, and then a cell strainer was used to filter out the debris. Red blood cells were lysed in an RBC lysis buffer (Thermo Fisher Scientific, Waltham, MA, USA). Splenocytes and lymph node cells were then suspended in a Roswell Park Memorial Institute (RPMI) culture medium containing 2% fetal bovine serum. To minimize the nonspecific binding of antibodies to FcR-bearing cells, a 2.4G2 hybridoma supernatant was used to pretreat these splenocytes and lymph node cells. After the 2.4G2 supernatant was removed by centrifugation, the cell surface molecules were stained with specific antibodies for 10 min and then washed twice with a staining buffer (1× PBS, 2% horse serum, and 0.05% NaN_3_). Finally, all cells were resuspended in a staining buffer and analyzed using a CytoFLEX flow cytometer (Beckman Coulter, Brea, CA, USA). The following antibodies were used in conjugation with a fluorochrome from BioLegend: CD3-FITC (2C11), CD8α-APC-Cy7 (53-6.7), CD19-PB (6D5), CD4-PE-Cy7 (GK1.5), and CD69-PE (H1.2F3). Positive cells were validated through comparison with the no-antibody staining control.

### 2.9. In Vitro Splenocyte Stimulation Assay

Splenocytes were prepared as described in Section 2.7. Cells were suspended in an RPMI culture medium containing 10% fetal bovine serum. The trypan blue exclusion assay was used to count the number of viable splenocytes. Subsequently, 5 × 10^5^ viable splenocytes were stimulated with ST7 (5 × 10^4^ U/mL), poly I:C (100 μg/mL), or both (poly I:C+ST7) in a 96-well plate containing culture medium for 24 h. The culture supernatant was then collected, and the IFN-γ level was determined using ELISA in accordance with standard protocols.

### 2.10. Intracellular IFN-γ Detection Assay

A total of 2 × 10^6^ splenocytes were incubated with phorbol myristate acetate (100 ng/mL) and an ionophore (A23187, 1 μg/mL) in a 0.5 mL RPMI culture medium containing 10% fetal bovine serum for 3 h. Brefeldin A (5 μg/mL) was added at the last 2 h of culture. The cells were first stained with antibodies for T-cell-related surface markers and Fixable Viability Dye eFluor 506 (Thermo Fisher Scientific) and then fixed by a Foxp3 Fix/Perm Buffer Set in accordance with standard protocols (BioLegend). Finally, the cells were intracellularly stained with an IFN-γ-APC (XMG1.2) antibody or an isotype control antibody for 20 min and were analyzed using flow cytometry.

### 2.11. Statistical Analysis

For data comparison, Mann–Whitney *t* tests and one-way analysis of variance were conducted using GraphPad Prism software (GraphPad, La Jolla, CA, USA). The error bars in the results represent the standard errors of the mean. Microbiota enrichment analysis was conducted using the linear discriminant analysis (LDA) effect size method. Data were compared using the Kruskal–Wallis and Wilcoxon tests. Differences were regarded as statistically significant at *p* ≤ 0.05, with a logarithmic LDA score of ≥2 [32].

## 3. Results

### 3.1. Effects of ST7 Treatment on Poly I:C-Induced Intestinal Damage and Weight Loss

**Figure 1:**
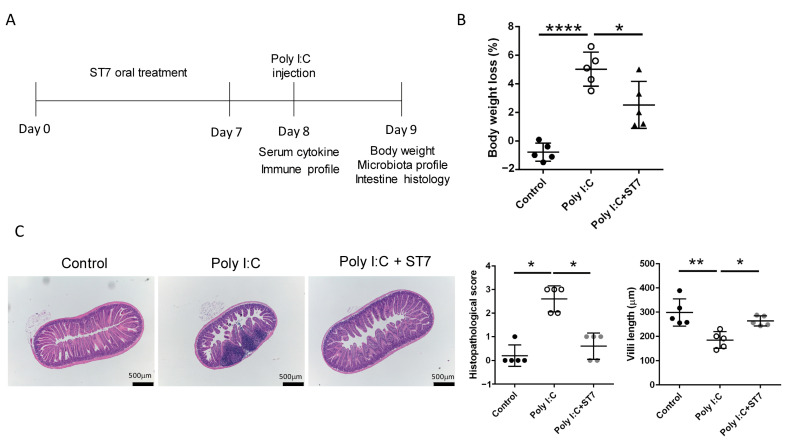
*Streptococcus thermophilus* strain 7 (ST7) treatment prevents small intestine injury induced by polyinosinic-polycytidylic acid (poly I:C). (**A**) Timeline of the ST7 experiments. The mice received ST7 orally for 7 days. On day 8, they were intraperitoneally injected with poly I:C for 2 h, and then their sera, spleens, and lymph nodes were harvested to examine the cytokine and immune cell activation profiles. Data pertaining to weight, microbiota, and intestinal histology were collected 24 h after poly I:C stimulation. (**B**) Quantification of the percentage of weight loss in mice treated with poly I:C and poly I:C + ST7. *n* = 5, * *p* < 0.05. **** *p* < 0.0001. (**C**) Representative photographs of H&E-stained paraffin-embedded sections from mice treated with poly I:C and poly I:C + ST7. The histopathological scores and villus-to-crypt ratios were quantified in the graph. *n* = 5, * *p* < 0.05, ** *p* < 0.01.

To determine the overall health condition and intestinal alterations following the administration of poly I:C, changes in weight and intestinal histology were evaluated. The results revealed that the administration of poly I:C resulted in a considerable reduction in weight (Figure 1B) and induced intestinal tissue inflammation with mild mucosal erosion (Figure 1C). ST7 treatment also considerably prevented weight loss, improved the histopathological score of the intestine (Figure 1B,C), and prevented intestinal villus length reductions induced by poly I:C stimulation. Taken together, these results revealed that ST7 treatment prevented TLR3-induced intestinal damage.

### 3.2. Effects of ST7 Treatment on the Serum Cytokine Profile after Poly I:C Stimulation

**Figure 2:**
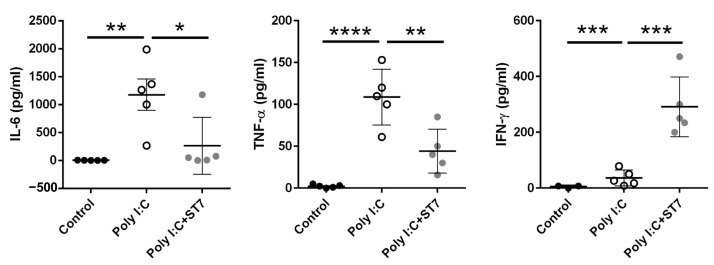
ST7 treatment modulates serum proinflammatory cytokine levels after poly I:C stimulation. Serum samples were collected from control (PBS-treated), poly I:C-treated, and poly I:C+ST7-treated mice. Serum IL-6, TNF-α, and IFN-γ levels were measured after poly I:C stimulation for 2 h. * *p* < 0.05, ** *p* < 0.01, *** *p* < 0.001, **** *p* < 0.0001.

We next evaluated the systemic inflammation level by determining the serum inflammatory cytokine levels. The intraperitoneal administration of poly I:C increased serum IL-6 and TNF-α levels (Figure 2), and these levels were substantially low in the ST7 treatment group (poly I:C + ST7). IFN-γ levels were also considerably increased in the poly I:C group. Notably, ST7 treatment synergistically increased the serum IFN-γ level (Figure 2).

### 3.3. Effects of ST7 Treatment on Intestinal IL-15 Expression in Poly I:C-Treated Mice

**Figure 3:**
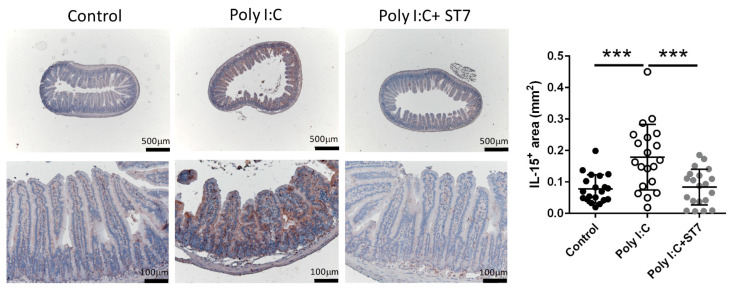
ST7 treatment prevents the upregulation of IL-15 in the intestinal villi after poly I:C stimulation. The expression of IL-15 in the intestinal villi was detected using immunohistochemistry. *n* = 5 per group. The graph depicts the quantification of the IL-15^+^ area in the small intestine. Four coronal sections were obtained from each mouse. Each dot indicates the IL-15^+^ area in each coronal section from the small intestines of mice. *** *p* < 0.001.

To determine how ST7 treatment prevented poly I:C-induced intestinal injury, we examined the expression of IL-15 in the intestine by using an immunohistochemical assay. Generally, IEC-derived IL-15 promotes the poly I:C-enhanced cytotoxicity of intraepithelial lymphocytes (IELs), which in turn results in intestinal injury [5]. Our data indicated that poly I:C considerably increased the expression of IL-15 in the IECs of the intestinal villi. In addition, ST7 treatment substantially prevented the upregulation of poly I:C-stimulated IL-15 expression in IECs (Figure 3).

### 3.4. Effects of ST7 Treatment on the Microbiota Profile of Poly I:C-Treated Mice

**Figure 4:**
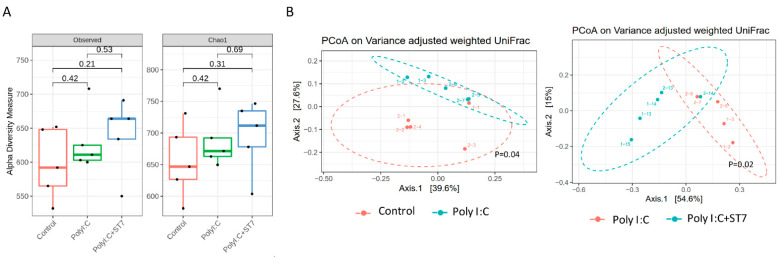
ST7 treatment modulates the distribution of the fecal microbiome after poly I:C stimulation. Fecal microbiome profiling was performed using high-throughput sequencing of the 16S ribosomal ribonucleic acid gene. (**A**) Alpha diversity of fecal microbiota and (**B**) principal coordinate analysis plots (obtained using variance-adjusted weighted UniFrac analysis) for the control, poly I:C, and poly I:C+ST7 mice. Permutational multivariate analysis of variance (vegan::adonis, 1000 permutations) revealed a considerable difference in beta diversity, which was quantified using the betadisper function (vegan::betadisper, 1000 permutations). Both adonis and betadisper indices yielded *p* values of <0.05 and >0.05, respectively.

**Figure 5:**
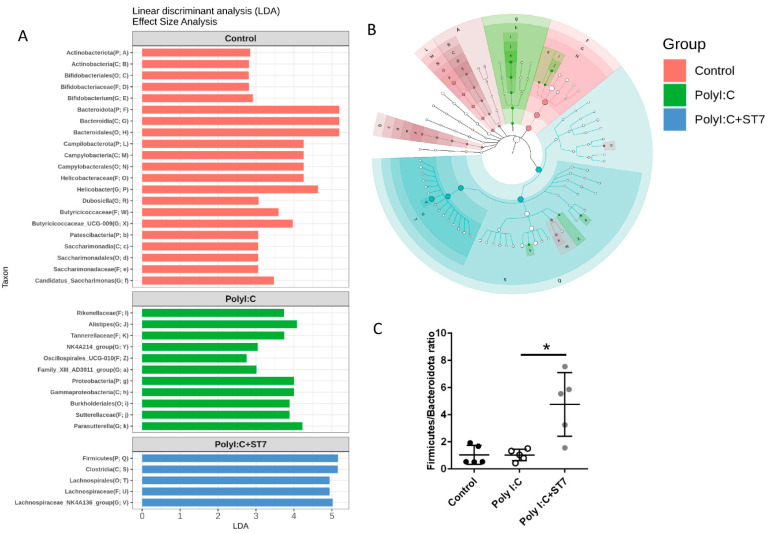
ST7 treatment alters the microbiota profile after poly I:C stimulation. (**A**) Linear discriminant analysis (LDA) for comparing the gut microbiota effect sizes of the control, poly I:C, and poly I:C+ST7 mice. Strong biomarkers were defined as taxa with an LDA score (log_10_) of ≥2. (**B**) Substantial taxa differences in the control, poly I:C, and poly I:C+ST7 mice are highlighted on the cladogram. (**C**) Ratio of Firmicutes to Bacteroidota in the control, poly I:C, and poly I:C+ST7 mice. * *p* < 0.01.

Inflammation and infection are accompanied by gut dysbiosis [33]. In this study, we examined whether ST7 can modulate the gut microbiota profile induced by poly I:C-induced inflammation. The results indicated that the alpha diversity of the fecal microbiota in the poly I:C treatment group was similar to that in the control group (Figure 4A). Evaluation of beta diversity through PCoA in variance-adjusted weighted UniFrac analysis revealed that, compared with the control group, the poly I:C treatment group had a considerably altered fecal microbiota profile (Figure 5B, left panel). Figure 5A depicts the substantial between-group differences in the abundance of microbiota species. Compared with the poly I:C treatment group, the control group had considerably higher LDA scores for Actinobacteriota, Bacteroidota, Campylobacterota, and Patescibacteria at the phylum level. In addition, compared with the control group, the poly I:C treatment group had higher LDA scores for Proteobacteria at the phylum level, Gammaproteobacteria at the class level, and *Alistipes* at the genus level. After 1 week of ST7 treatment and subsequent poly I:C stimulation, the fecal microbiota profile was substantially different from that of the poly I:C treatment group (Figure 4B, right panel). However, no considerable difference was detected in alpha diversity (Figure 4A). The LDA scores for Firmicutes at the phylum level, Clostridia at the class level, and Lachnospirales at the order level in the mice that received ST7 treatment and poly I:C stimulation (poly I:C + ST7) were higher than those of the poly I:C-treated control group (Figure 5A). In an inflammatory intestinal disease, a decreased Firmicutes-to-Bacteroidetes ratio is observed [34]. In this study, this ratio was considerably increased by ST7 treatment in the poly I:C treatment group (Figure 5C). Taken together, these results indicate that ST7 treatment can ameliorate dysbiosis in mice with poly I:C-induced intestinal injury.

### 3.5. Effects of ST7 Treatment on T-Cell Activation after Poly I:C Stimulation

**Figure 6:**
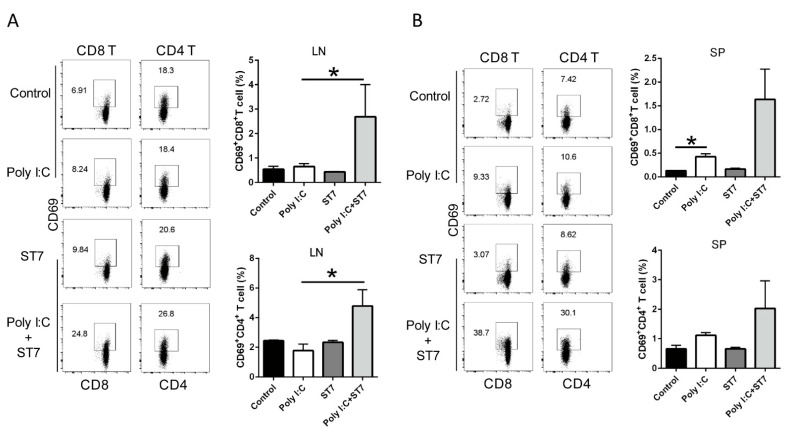
ST7 treatment promotes the activation of T cells after poly I:C stimulation. (**A**) The expression of CD69 by CD8^+^ T and CD4^+^ T cells in the mesenteric lymph nodes (LNs) and (**B**) spleen (SP) after 2 h of poly I:C stimulation was analyzed using flow cytometry. The percentages of CD69^+^CD8^+^ T and CD69^+^CD4^+^ T cells in LNs and SP are depicted as bar graphs. Data are presented as mean ± standard error of the mean (SEM). *n* = 5 per group, * *p* < 0.05.

We next investigated whether ST7 treatment modulates T-cell activation by examining CD69 expression after poly I:C stimulation. The results revealed that neither poly I:C stimulation nor ST7 treatment affected the percentages of CD69^+^CD8^+^T and CD69^+^CD4^+^ T cells in the mesenteric lymph nodes. However, after ST7 treatment for 7 days, the percentages of CD69^+^CD8^+^ T and CD69^+^CD4^+^ T cells increased after 2 h of poly I:C stimulation (Figure 6A). In the spleen, poly I:C stimulation considerably increased the percentage of CD69^+^CD8^+^T cells and slightly increased the percentage of CD69^+^CD4^+^ T-cells (Figure 6B). ST7 treatment also considerably increased the percentages of CD69^+^CD8^+^ and CD69^+^CD4^+^ T cells in the spleen after 2 h of poly I:C stimulation (Figure 6B). These results indicated that ST7 treatment can promote T-cell activation upon TLR3 activation.

### 3.6. Effects of ST7 Treatment on T-Cell Activation and IFN-γ Production after Poly I:C Stimulation

**Figure 7:**
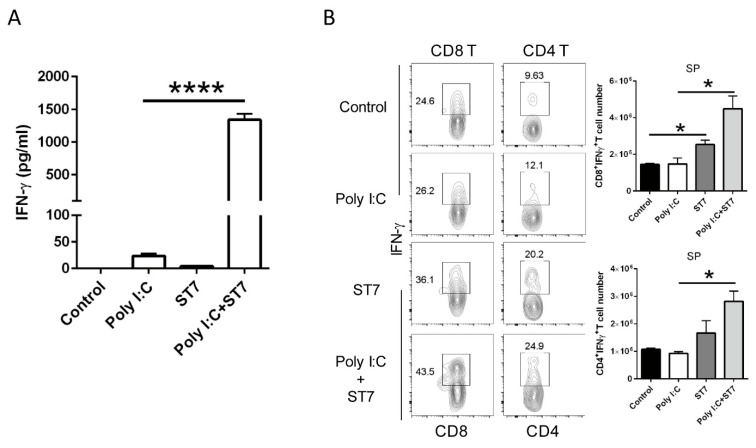
ST7 treatment increases the IFN-γ-induced production of T cells after poly I:C stimulation. (**A**) Splenocytes were incubated with poly I:C, ST7, and poly I:C + ST7 for 24 h. IFN-γ was then quantified in the culture supernatant through an enzyme-linked immunosorbent assay. *n* = 3, **** *p* < 0.001. (**B**) The expression of IFN-γ by CD8^+^ T and CD4^+^ T cells in the spleen (SP) after 2 h of poly I:C stimulation was analyzed using flow cytometry. The CD8^+^IFN-γ^+^ T and CD4^+^IFN-γ^+^ T cell counts in the SP are represented as bar graphs. Data are represented as mean ± SEM. *n* = 5 per group, * *p* < 0.05.

IFN-γ is a critical effector cytokine that induces an antiviral immune response. In this study, because ST7 treatment increased the serum IFN-γ level upon TLR3 activation, we quantified IFN-γ levels in T cells after poly I:C stimulation. In vitro splenocyte stimulation revealed that ST7 significantly increased the production of IFN-γ from splenocytes upon poly I:C stimulation (Figure 7A). In addition, the results of the oral heat-inactivated ST7 experiment revealed that in vivo poly I:C stimulation for 2 h did not affect IFN-γ levels in CD8^+^ T and CD4^+^ T cells. ST7 treatment for 7 days led to the upregulation of IFN-γ expression in splenic CD8 and CD4 T cells not only directly (Figure 7B, control vs. ST7) but also upon poly I:C stimulation (Figure 7B, poly I:C vs. poly I:C + ST7). Taken together, these results indicated that ST7 treatment can promote T-cell effector function.

## 4. Discussion

The heat-inactivated *S. thermophilus* possess an immunomodulatory function. In this study, we discovered that, in a poly I:C-induced intestinal damage mouse model, heat-inactivated ST7 treatment modulated the host fecal microbiota and inflammatory response. The results indicated that oral ST7 treatment effectively prevented poly I:C-induced injury to the intestinal epithelium by preventing the upregulation of serum inflammatory cytokines (TNF-α and IL-6) and IL-15 in IECs. In addition, ST7 treatment enhanced host antiviral activity by enhancing T-cell activation and effector function under poly I:C stimulation conditions. It also substantially altered the gut microbiota profile; that is, it prevented the upregulation of inflammation-associated Gammaproteobacteria and *Alistipes* and increased the levels of Firmicutes in the fecal microbiota after poly I:C stimulation. Taken together, our findings indicated that ST7 prevented the inflammatory response, enhanced the T-cell effector function, and modulated the microbiota profile of mice with poly I:C-induced intestinal injury.

Intestinal inflammation and infection are accompanied by an imbalance in the gut microbiota [33]. Viral infections elicit an inflammatory response, resulting in gut barrier dysfunction [35]. However, the mechanism through which a dsRNA virus infection affects the gut microbiome remains unclear. In this study, to mimic a dsRNA viral infection, we used intraperitoneal poly I:C injections, which resulted in substantial gut microbial dysbiosis and increased the number of Gammaproteobacteria and *Alistipes* in the fecal microbiota; these alterations are associated with inflammatory responses in colitis and hypertension, respectively [36,37]. Notably, the numbers of Gammaproteobacteria in the ST7-treated mice were not higher than those in the poly I:C-treated mice. ST7 treatment considerably increased the numbers of Firmicutes and *Clostridium* species in the poly I:C-treated mice. Firmicutes have anti-inflammatory effects and can alleviate the progression of inflammatory bowel disease [34]. The *Clostridium* species positively regulates the differentiation, function, and accumulation of T cells in the mouse colon, providing anti-inflammatory and protective effects [38]. Collectively, our data suggest that the oral administration of heat-inactivated ST7 can prevent the dysbiosis of microbiota by modulating the proinflammatory microbiota profile toward anti-inflammatory properties upon dsRNA viral stimulation. According to a previous study, the intestinal dysbiosis profile of a TLR4-induced sepsis animal model differs from that of a TLR3-induced intestinal injury model [16]. In addition, intraperitoneal LPS treatment considerably decreases the numbers of *Fusobacterium* in the fecal microbiota, and oral live *S. thermophiles* treatment considerably increases the numbers of *Fusobacterium* after the administration of LPS [16]. However, in this study, the numbers of *Fusobacterium* were not affected by either poly I:C or poly I:C + ST7 treatment. Taken together, these data indicate that *S. thermophiles* can modulate different types of TLR-induced microbial dysbiosis.

Strains of *S. thermophilus* can modulate immune responses, with each strain exerting either a proinflammatory or an anti-inflammatory effect [16,39]. In a TLR4-induced sepsis animal model, treatment with *S. thermophiles* alleviates intestinal injury and decreases the serum levels of inflammatory cytokines (IL-6 and TNF-α) [16]. This finding is in line with that of our study, in which we reported that *S. thermophilus* bacteria exhibit an anti-inflammatory property. Although ST7 treatment increased serum IFN-γ amounts and IFN-γ can potentiate pro-inflammatory signaling [40], ST7 can differentially affect inflammatory cytokines (IL-6 and TNF-α) and IFN-γ production after 2 h poly I:C stimulation. Upon poly I:C stimulation, oral ST7 treatment prevents IL-15 overexpression in the IECs, thereby potentially protecting the intestine from the detrimental poly I:C-induced immune response. After 2 h of poly I:C stimulation, ST7 treatment enhances not only the activation of CD8 and CD4 T cells but also their IFN-γ production. IFN-γ can promote the type I interferons, including IFN-α and IFN-β, which can antagonize virus replication [9,41]. These findings suggest that ST7 enhances T-cell effector function during the early stage of viral infection. Taken together, these findings imply that ST7 can prevent TLR3-induced inflammation and enhance the T-cell immune response during the early stage of viral infection.

Numerous studies have indicated that both viable and heat-inactivated lactic acid bacteria can modulate the microbiome. For instance, gut microbial co-occurrence network analysis has revealed that live and heat-inactivated *Lactiplantibacillus plantarum* can reorganize human gut microbial community structures [42]. Another animal study demonstrated that viable and heat-inactivated *Lactobacillus plantarum* can reduce hypercholesterolemia and regulate the intestinal microbiota and metabolites related to lipid metabolism [43]. In addition, various strains of heat-killed lactic acid bacteria (*S. thermophilus*, *L. bulgaricus*, and *L. acidophilus*) can disrupt the expression of the tight junction proteins ZO-1 and occludin and prevent epithelial barrier dysfunction induced by inflammatory cytokines [44]. However, although live *S. thermophilus* can modulate intestinal dysbiosis in mice with sepsis [16], whether and how nonviable *S. thermophilus* modulates the intestinal microbiome remain unclear. In this study, heat-inactivated ST7 pretreatment prevented epithelial barrier dysfunction and prevented the increase in TNF-α levels in the serum after poly I:C stimulation. This anti-inflammatory effect can protect IECs against inflammatory-cytokine-induced dysfunction and IL-15-induced IEC and IEL cytotoxicity [45]. However, the mechanism through which ST7 functions remains unclear and warrants further investigation.

## 5. Conclusions

Heat-inactivated ST7 treatment has an immunomodulatory property, prevents detrimental inflammatory responses in the intestine, and modulates gut microbial dysbiosis. ST7 treatment also prevents the upregulation of inflammatory cytokines (IL-6 and TNF-α) in the serum and the expression of IL-15 in the IECs and promotes the activation of T cells and their IFN-γ production. In addition, ST7 treatment prevents the upregulation of inflammation-associated Gammaproteobacteria and *Alistipes* and increases the numbers of Firmicutes in the fecal microbiota after poly I:C stimulation. This finding indicates that heated-inactivated ST7 can be used to prevent injuries resulting from dsRNA-virus-induced intestinal inflammation, which in turn may prevent intestinal dysfunction due to gastroenteritis. Regarding the limitations of this study, the biological components of heat-inactivated ST7 that are responsible for these anti-inflammatory and immune modulation activities remain unclear, and the underlying mechanisms require extensive investigations.

## Data Availability

The data sets used and/or analyzed in this study are available from the corresponding authors upon reasonable request.

## References

[1] Crawford S.E., Ramani S., Tate J.E., Parashar U.D., Svensson L., Hagbom M., Franco M.A., Greenberg H.B., O’Ryan M., Kang G. (2017). Rotavirus infection. Nat. Rev. Dis. Prim..

[2] Petri W.A., Miller M., Binder H.J., Levine M.M., Dillingham R., Guerrant R.L. (2008). Enteric infections, diarrhea, and their impact on function and development. J. Clin. Invest..

[3] Guerrero C.A., Acosta O. (2016). Inflammatory and oxidative stress in rotavirus infection. World J. Virol..

[4] Alexopoulou L., Holt A.C., Medzhitov R., Flavell R.A. (2001). Recognition of double-stranded RNA and activation of NF-kappaB by Toll-like receptor 3. Nature.

[5] Zhou R., Wei H., Sun R., Tian Z. (2007). Recognition of double-stranded RNA by TLR3 induces severe small intestinal injury in mice. J. Immunol..

[6] Tada A., Zelaya H., Clua P., Salva S., Alvarez S., Kitazawa H., Villena J. (2016). Immunobiotic Lactobacillus strains reduce small intestinal injury induced by intraepithelial lymphocytes after Toll-like receptor 3 activation. Inflamm. Res..

[7] Zhao H.W., Yue Y.H., Han H., Chen X.L., Lu Y.G., Zheng J.M., Hou H.T., Lang X.M., He L.L., Hu Q.L. (2017). Effect of toll-like receptor 3 agonist poly I:C on intestinal mucosa and epithelial barrier function in mouse models of acute colitis. World J. Gastroenterol..

[8] Ruder B., Atreya R., Becker C. (2019). Tumour Necrosis Factor Alpha in Intestinal Homeostasis and Gut Related Diseases. Int. J. Mol. Sci..

[9] Rosendahl Huber S., van Beek J., de Jonge J., Luytjes W., van Baarle D. (2014). T cell responses to viral infections–opportunities for Peptide vaccination. Front. Immunol..

[10] Glenwright A.J., Pothula K.R., Bhamidimarri S.P., Chorev D.S., Basle A., Firbank S.J., Zheng H., Robinson C.V., Winterhalter M., Kleinekathofer U. (2017). Structural basis for nutrient acquisition by dominant members of the human gut microbiota. Nature.

[11] Hand T.W., Vujkovic-Cvijin I., Ridaura V.K., Belkaid Y. (2016). Linking the Microbiota, Chronic Disease, and the Immune System. Trends Endocrinol. Metab..

[12] Soderholm A.T., Pedicord V.A. (2019). Intestinal epithelial cells: At the interface of the microbiota and mucosal immunity. Immunology.

[13] Chiang S.S., Pan T.M. (2012). Beneficial effects of Lactobacillus paracasei subsp. paracasei NTU 101 and its fermented products. Appl. Microbiol. Biotechnol..

[14] Garcia-Castillo V., Komatsu R., Clua P., Indo Y., Takagi M., Salva S., Islam M.A., Alvarez S., Takahashi H., Garcia-Cancino A. (2019). Evaluation of the Immunomodulatory Activities of the Probiotic Strain Lactobacillus fermentum UCO-979C. Front. Immunol..

[15] Bomko T.V., Nosalskaya T.N., Kabluchko T.V., Lisnyak Y.V., Martynov A.V. (2017). Immunotropic aspect of the Bacillus coagulans probiotic action. J. Pharm. Pharm..

[16] Han F., Wu G., Zhang Y., Zheng H., Han S., Li X., Cai W., Liu J., Zhang W., Zhang X. (2020). Streptococcus thermophilus Attenuates Inflammation in Septic Mice Mediated by Gut Microbiota. Front. Microbiol..

[17] Preidis G.A., Hill C., Guerrant R.L., Ramakrishna B.S., Tannock G.W., Versalovic J. (2011). Probiotics, enteric and diarrheal diseases, and global health. Gastroenterology.

[18] Pique N., Berlanga M., Minana-Galbis D. (2019). Health Benefits of Heat-Killed (Tyndallized) Probiotics: An Overview. Int. J. Mol. Sci..

[19] Chuang L., Wu K.G., Pai C., Hsieh P.S., Tsai J.J., Yen J.H., Lin M.Y. (2007). Heat-killed cells of lactobacilli skew the immune response toward T helper 1 polarization in mouse splenocytes and dendritic cell-treated T cells. J. Agric. Food Chem..

[20] Sule J., Korosi T., Hucker A., Varga L. (2014). Evaluation of culture media for selective enumeration of bifidobacteria and lactic acid bacteria. Braz. J. Microbiol..

[21] Erben U., Loddenkemper C., Doerfel K., Spieckermann S., Haller D., Heimesaat M.M., Zeitz M., Siegmund B., Kuhl A.A. (2014). A guide to histomorphological evaluation of intestinal inflammation in mouse models. Int. J. Clin. Exp. Pathol..

[22] Tanaka-Okamoto M., Itoh Y., Miyoshi J., Mizoguchi A., Mizutani K., Takai Y., Inoue M. (2014). Genetic ablation of afadin causes mislocalization and deformation of Paneth cells in the mouse small intestinal epithelium. PLoS ONE.

[23] Ma L.J., Acero L.F., Zal T., Schluns K.S. (2009). Trans-presentation of IL-15 by intestinal epithelial cells drives development of CD8alphaalpha IELs. J. Immunol..

[24] Lee G.A., Lin T.N., Chen C.Y., Mau S.Y., Huang W.Z., Kao Y.C., Ma R.Y., Liao N.S. (2018). Interleukin 15 blockade protects the brain from cerebral ischemia-reperfusion injury. Brain Behav. Immun..

[25] Lee G.A., Lin Y.K., Lai J.H., Lo Y.C., Yang Y.S.H., Ye S.Y., Lee C.J., Wang C.C., Chiang Y.H., Tseng S.H. (2021). Maternal Immune Activation Causes Social Behavior Deficits and Hypomyelination in Male Rat Offspring with an Autism-Like Microbiota Profile. Brain Sci..

[26] Callahan B.J., McMurdie P.J., Rosen M.J., Han A.W., Johnson A.J., Holmes S.P. (2016). DADA2: High-resolution sample inference from Illumina amplicon data. Nat. Methods.

[27] Callahan B.J., Sankaran K., Fukuyama J.A., McMurdie P.J., Holmes S.P. (2016). Bioconductor Workflow for Microbiome Data Analysis: From raw reads to community analyses. F1000Res.

[28] Quast C., Pruesse E., Yilmaz P., Gerken J., Schweer T., Yarza P., Peplies J., Glockner F.O. (2013). The SILVA ribosomal RNA gene database project: Improved data processing and web-based tools. Nucleic Acids Res..

[29] Schliep K.P. (2011). phangorn: Phylogenetic analysis in R. Bioinformatics.

[30] McMurdie P.J., Holmes S. (2013). phyloseq: An R package for reproducible interactive analysis and graphics of microbiome census data. PloS one.

[31] Chen J., Bittinger K., Charlson E.S., Hoffmann C., Lewis J., Wu G.D., Collman R.G., Bushman F.D., Li H.Z. (2012). Associating microbiome composition with environmental covariates using generalized UniFrac distances. Bioinformatics.

[32] Segata N., Izard J., Waldron L., Gevers D., Miropolsky L., Garrett W.S., Huttenhower C. (2011). Metagenomic biomarker discovery and explanation. Genome Biol..

[33] Khosravi A., Yanez A., Price J.G., Chow A., Merad M., Goodridge H.S., Mazmanian S.K. (2014). Gut microbiota promote hematopoiesis to control bacterial infection. Cell Host Microbe.

[34] Stojanov S., Berlec A., Strukelj B. (2020). The Influence of Probiotics on the Firmicutes/Bacteroidetes Ratio in the Treatment of Obesity and Inflammatory Bowel disease. Microorganisms.

[35] Lian S., Liu J., Wu Y., Xia P., Zhu G. (2022). Bacterial and Viral Co-Infection in the Intestine: Competition Scenario and Their Effect on Host Immunity. Int. J. Mol. Sci..

[36] Parker B.J., Wearsch P.A., Veloo A.C.M., Rodriguez-Palacios A. (2020). The Genus Alistipes: Gut Bacteria with Emerging Implications to Inflammation, Cancer, and Mental Health. Front. Immunol..

[37] Naqvi S., Asar T.O., Kumar V., Al-Abbasi F.A., Alhayyani S., Kamal M.A., Anwar F. (2021). A cross-talk between gut microbiome, salt and hypertension. Biomed. Pharm..

[38] Atarashi K., Tanoue T., Oshima K., Suda W., Nagano Y., Nishikawa H., Fukuda S., Saito T., Narushima S., Hase K. (2013). Treg induction by a rationally selected mixture of Clostridia strains from the human microbiota. Nature.

[39] Kekkonen R.A., Kajasto E., Miettinen M., Veckman V., Korpela R., Julkunen I. (2008). Probiotic Leuconostoc mesenteroides ssp. cremoris and Streptococcus thermophilus induce IL-12 and IFN-gamma production. World J. Gastroenterol..

[40] De Simoni M.G., Terreni L., Chiesa R., Mangiarotti F., Forloni G.L. (1997). Interferon-gamma potentiates interleukin (IL)-6 and tumor necrosis factor-alpha but not IL-1beta induced by endotoxin in the brain. Endocrinology.

[41] Lee A.J., Ashkar A.A. (2018). The Dual Nature of Type I and Type II Interferons. Front Immunol..

[42] Lee C.C., Liao Y.C., Lee M.C., Cheng Y.C., Chiou S.Y., Lin J.S., Huang C.C., Watanabe K. (2022). Different Impacts of Heat-Killed and Viable Lactiplantibacillus plantarum TWK10 on Exercise Performance, Fatigue, Body Composition, and Gut Microbiota in Humans. Microorganisms.

[43] Li Y., Chen M., Ma Y., Yang Y., Cheng Y., Ma H., Ren D., Chen P. (2022). Regulation of viable/inactivated/lysed probiotic Lactobacillus plantarum H6 on intestinal microbiota and metabolites in hypercholesterolemic mice. NPJ Sci. Food.

[44] Kaur H., Ali S.A. (2022). Probiotics and gut microbiota: Mechanistic insights into gut immune homeostasis through TLR pathway regulation. Food Funct..

[45] Villena J., Vizoso-Pinto M.G., Kitazawa H. (2016). Intestinal Innate Antiviral Immunity and Immunobiotics: Beneficial Effects against Rotavirus Infection. Front. Immunol..

